# Motion sensitive network for action recognition in control and decision-making of autonomous systems

**DOI:** 10.3389/fnins.2024.1370024

**Published:** 2024-03-25

**Authors:** Jialiang Gu, Yang Yi, Qiang Li

**Affiliations:** Computer Science and Engineering, Sun Yat-sen University, Guangdong, China

**Keywords:** deep learning, action recognition, computer vision, visual perception, motion information, spatial-temporal feature, practical application

## Abstract

Spatial-temporal modeling is crucial for action recognition in videos within the field of artificial intelligence. However, robustly extracting motion information remains a primary challenge due to temporal deformations of appearances and variations in motion frequencies between different actions. In order to address these issues, we propose an innovative and effective method called the Motion Sensitive Network (MSN), incorporating the theories of artificial neural networks and key concepts of autonomous system control and decision-making. Specifically, we employ an approach known as Spatial-Temporal Pyramid Motion Extraction (STP-ME) module, adjusting convolution kernel sizes and time intervals synchronously to gather motion information at different temporal scales, aligning with the learning and prediction characteristics of artificial neural networks. Additionally, we introduce a new module called Variable Scale Motion Excitation (DS-ME), utilizing a differential model to capture motion information in resonance with the flexibility of autonomous system control. Particularly, we employ a multi-scale deformable convolutional network to alter the motion scale of the target object before computing temporal differences across consecutive frames, providing theoretical support for the flexibility of autonomous systems. Temporal modeling is a crucial step in understanding environmental changes and actions within autonomous systems, and MSN, by integrating the advantages of Artificial Neural Networks (ANN) in this task, provides an effective framework for the future utilization of artificial neural networks in autonomous systems. We evaluate our proposed method on three challenging action recognition datasets (Kinetics-400, Something-Something V1, and Something-Something V2). The results indicate an improvement in accuracy ranging from 1.1% to 2.2% on the test set. When compared with state-of-the-art (SOTA) methods, the proposed approach achieves a maximum performance of 89.90%. In ablation experiments, the performance gain of this module also shows an increase ranging from 2% to 5.3%. The introduced Motion Sensitive Network (MSN) demonstrates significant potential in various challenging scenarios, providing an initial exploration into integrating artificial neural networks into the domain of autonomous systems.

## 1 Introduction

With the rapid development of computer vision technology, action recognition in videos (Sun et al., 2022) has become a crucial challenge, finding applications in areas such as autonomous driving and virtual reality. In this context, video action recognition is not just an academic research field but a key component for addressing real-world problems and enhancing the intelligence of AI systems. Recently, action recognition methods based on convolutional neural networks (CNNs) have gained significant attention. Among them, 3D convolutional networks are renowned for directly extracting spatiotemporal features from videos, but they suffer from high computational costs, limiting their efficiency for human action recognition. On the other hand, 2D convolutional networks (Yao et al., 2022), especially two-stream networks, extract motion information by capturing multimodal cues. However, fusing multimodal information still poses challenges, and the pre-computation of optical flow is computationally expensive (Alayrac et al., 2022; Islam et al., 2023). In recent years, successful approaches have emerged by extracting motion features from RGB using embeddable modules within 2D convolutional networks, achieving satisfactory performance at a lower cost (Wu et al., 2020). Although these modules capture some motion features, they may overlook spatial scale variations over time and inconsistent action frequencies across different actions. This motivates us to propose a novel approach aimed at handling spatiotemporal features in video action recognition more comprehensively and efficiently.

This paper explores the utilization of artificial neural networks (ANNs) in the context of spatial and temporal modeling, contributing to the theoretical foundations and practical applications of ANNs in autonomous system control and decision-making. However, applications in the field of ubiquitous Human Activity Recognition (HAR) have been relatively limited. To address the issue of information loss during channel compression, researchers have proposed a multi-frequency channel attention framework based on Discrete Cosine Transform (DCT) to better compress channels and utilize other frequency components (Xu et al., 2023). On the other hand, Federated Learning (FL) shows potential in HAR tasks, but the non-IID nature of sensor data poses challenges for traditional FL methods. To tackle this, researchers have introduced the ProtoHAR framework, which leverages global prototypes and personalized training to address representation and classifier issues in heterogeneous FL environments (Cheng et al., 2023). Additionally, wearable sensor-based HAR has gained significant attention, where the phenomenon of channel folding in existing methods impairs model generalization. Researchers have proposed a channel equalization method to balance feature representation by reactivating suppressed channels (Huang et al., 2022). These studies provide important references and guidance for the development and practical applications in the HAR field. In the realm of video-based action recognition (Zheng et al., 2022a), complexities arise from the need to handle intricate data distributions and extract both spatial and temporal information concurrently. Distinguishing diverse action classes, addressing scale changes, and accommodating inconsistent action frequency require sophisticated spatial and temporal modeling (Cob-Parro et al., 2024). For instance, discerning actions like "Running" from “Walking” involves not only recognizing visual tempo differences but also understanding spatial scale variations. Similarly, “Brush Teeth” and “Apply Eye Makeup” have great differences in spatial scale despite sharing high similarities in the temporal dimension (Kulsoom et al., 2022). Learning the intention of human action from such data in videos poses a great challenge (Zheng et al., 2024). In certain scenarios, fine-grained recognition of actions becomes crucial, requiring more detailed spatial and temporal modeling. Learning the intent behind human behavior from such data in videos poses a significant challenge. Additionally, there are substantial challenges in the fusion of multimodal information, especially when it involves additional modalities such as optical flow. Existing methods face difficulties in effectively integrating different modalities, and the computational cost of pre-computing modalities like optical flow remains a bottleneck. Similarly, modeling actions in long-term videos often encounters challenges related to memory. Models may struggle to capture the evolution of actions over long time spans and maintain consistent understanding throughout the entire sequence. Imbalance and scarcity of samples across different action categories in the dataset present another problem, as the models may exhibit bias when learning minority class actions, thereby affecting overall performance. In some application scenarios, real-time requirements for action recognition models are high. For example, in autonomous driving systems, achieving high accuracy while ensuring fast inference speed to adapt to real-time environments is crucial (Lin and Xu, 2023). Meanwhile, action recognition models are susceptible to adversarial attacks, where subtle perturbations to the model inputs can lead to misclassification. Improving model adversarial robustness and resilience remains a challenge (Chen et al., 2019).

In this paper, we propose a new approach called Motion Sensitive Network (MSN) that addresses the challenge of efficiently recognizing complex actions with varying spatial scales and visual tempos. To achieve this, we introduce two new modules: the Temporal Spatial Pyramid Motion Extraction (STP-ME) module and the Deformable Scales Motion Excitation (DS-ME) module. The STP-ME module extracts implicit motion information by taking consecutive frames as input and using feature difference to focus on the position and tempo of the action occurring between frames. This information is incorporated into the single RGB frame (Liu et al., 2021), allowing for better alignment of the temporal and spatial dimensions at different scales. The DS-ME module addresses irregular deformation of the action subject in space and long-range feature alignment issues. It uses multiscale deformable convolutions to model the complete action region (He and Tang, 2023), allowing for more accurate representation of different motion splits. Additionally, to address numerical problems with negative values, we use the absolute value of the feature. Overall, our framework can be broken down into three steps: extracting effective motion information in the early stage, giving higher weight to motion features in the later stage, and doing numerical processing to avoid harmful results during processing (Luo, 2023). Our proposed MSN method effectively handles the challenges of action recognition, improving on existing 2D and 3D CNN-based methods. By leveraging ANNs in spatial and temporal modeling, this work contributes to enhancing the theoretical foundations and practical applications of ANNs in autonomous system control and decision-making.

The contributions of this paper can be summarized in the following three aspects:

(1) The paper introduces a novel approach known as the Motion Sensitive Network (MSN) for action recognition. This method is characterized by its simplicity and effectiveness in accurately estimating scale variations, thereby enhancing overall network performance in action recognition tasks.
(2) The paper proposes a unique Time-Space Pyramid Motion Extraction (STP-ME) module. This module leverages a pyramid structure to extract multi-scale temporal features, thereby fortifying the model's robustness across diverse action scenarios. The STP-ME module is designed to address challenges associated with scale variations and capture motion information across different time scales.
(3) The paper introduces the Variable Scale Motion Excitation (DS-ME) module as an innovative solution to challenges posed by unique and irregular motion patterns in dynamic scenes. This module utilizes deformable scale convolutions to adaptively modify the motion scale of target objects before computing temporal differences on consecutive frames. This approach aims to enhance the model's ability to handle objects with varying scales during motion.

The organizational structure of this paper is as follows: The introduction (Section 1) sets the stage by presenting the background, significance, and motivation for the research, highlighting challenges in existing action recognition methods, and outlining the contributions of the proposed Motion Sensitive Network (MSN). Section 2, “Relevant Work,” conducts a comprehensive review of existing literature, emphasizing prior research on motion sensitivity in action recognition and identifying gaps in current approaches. The third section, “Method,” provides a detailed exposition of the MSN architecture, elucidating its design principles and showcasing its motion-sensitive modules. Moving on to Section 4, “Experiment,” the paper delves into the experimental setup, detailing the datasets used, metrics employed for performance assessment, and the methodology for training MSN, while Section 5, “Discussion,” critically analyzes experimental results. This section interprets findings, assesses MSN's effectiveness in addressing motion sensitivity, and discusses potential applications and limitations. Finally, in Section 6, “Conclusion,” the paper synthesizes key discoveries, underscores the contributions made by MSN, discusses broader implications for the field of action recognition, and proposes avenues for future research. This organized structure guides readers through a coherent narrative (Han et al., 2022), facilitating a comprehensive understanding of the research from problem introduction to proposed solution, experimental validation, discussion, and ultimate conclusion.

## 2 Related work

The realm of action recognition within computer vision has undergone significant exploration (Zhang et al., 2022; Dai et al., 2023; Wu et al., 2023), with convolutional neural networks (CNNs) at the forefront of innovation (Xu et al., 2022). Two major categories, two-stream CNNs and 3D CNNs, have shaped the landscape. Next, we will delve into the theoretical foundations and practical applications of artificial neural networks in the field of autonomous system control and decision-making.

Simonyan and Zisserman (2014) proposed a multi-stream network for action recognition, consisting of two separate branches: a temporal convolutional network and a spatial convolutional network. Both branches have the same architecture, with the temporal stream learning motion features from stacked optical flows and the spatial stream extracting spatial features from still images (Wang et al., 2020). The two streams are then fused to obtain the final classification result. However, this approach has some drawbacks. Firstly, the computational cost is relatively high, particularly due to the complexity of optical flow computation. The stacking of optical flows may result in expensive computational overhead, especially when dealing with long video sequences or high frame-rate videos. Secondly, the method's reliance on optical flow makes it sensitive to video noise and motion blur, impacting the reliability of accurately extracting motion features. Additionally, the dependence on optical flow introduces sensitivity to video noise and motion blur, affecting the reliability of accurately extracting motion features. Moreover, the challenge of modal fusion is also a concern, as effective fusion requires careful design to ensure that features extracted from both streams collaborate without interference. Lastly, the method may have limitations in modeling spatiotemporal relationships, especially in complex motion scenarios, such as non-rigid motion or rapidly changing movements. This may result in constraints on the comprehensive capture of complex spatiotemporal dynamic relationships. Wang et al. (2016) proposed a Temporal Segment Network (TSN) based on the two-stream CNN, which utilizes a sparse time sampling strategy to randomly extract video fragments after time-domain segmentation. TSN addresses the insufficient modeling ability of long-range temporal structure in two-stream CNNs. However, this approach may have some potential limitations. Sparse temporal sampling strategies may result in the loss of crucial temporal information during the model training process, especially for modeling long-duration actions, which may not be adequately captured. Furthermore, this randomness in sampling may hinder the model's ability to effectively capture critical temporal patterns for specific types of actions, thereby impacting its performance. Building on TSN, Zhou et al. (2018) attempted to extract connections between video frames of different scales by convolving video frames of different lengths, performing multi-scale feature fusion, and obtaining behavior recognition results. However, applying convolution to video frames of different lengths may increase the computational complexity of the model in handling information at different scales, thereby impacting the training and inference efficiency of the model. He et al. (2019) proposed a local and global module to hierarchically model temporal information based on action category granularity, while Li et al. (2020) proposed motion excitation and multiple temporal aggregation modules to encode short- and long-range motion effectively and efficiently, integrated into standard ResNet blocks for temporal modeling. Wang et al. (2021) focused on capturing multi-scale temporal information for efficient action recognition, presenting a video-level motion modeling framework with a proposed temporal difference module for capturing short- and long-term temporal structure. However, these methods may share some potential common drawbacks. Firstly, approaches such as local and global modules based on action category granularity, hierarchical networks from coarse to fine, motion excitation, multiple temporal aggregation modules, video-level motion modeling frameworks, and temporal offset modules may require more complex network structures and additional parameters to achieve layered modeling of temporal information. This may lead to increased computational complexity, heightened training difficulty, and an increased demand for hardware resources. Secondly, these methods might necessitate carefully designed hyperparameters and model structures to adapt to different time scales and action categories. In practical applications, this could require extensive parameter tuning and model optimization, raising the method's usage threshold and operational difficulty. Additionally, these methods may encounter memory issues when dealing with long temporal video sequences in temporal modeling. The model might struggle to effectively capture the evolution of actions over extended time ranges and maintain consistent understanding throughout the entire sequence. When handling long temporal videos, these methods might need additional mechanisms to ensure the model's effectiveness and stability.

Another type of method attempts to learn spatio-temporal features directly from RGB frames using 3D CNNs. The 3D convolutional network for action recognition was introduced by Yang et al. (2019), which uses a 3D convolution kernel to perform 3D convolution on the input and directly extracts spatio-temporal features along the spatial and temporal dimensions of the video. Tran et al. (2018) constructed a C3D network framework using 3D convolution and 3D pooling operations. Carreira and Zisserman (2017) combined a two-stream network and a 3D CNN to propose an I3D network framework based on the inception-V1 model, using RGB and optical flow as inputs. Diba et al. (2018) and others improved the I3D by using different scales of convolution to build the TTL layer and using 3D-DenseNet as the basic network to build the T3D network framework. Qiu et al. (2019) and others proposed a P3D network, which uses 133 convolution and 311 convolutions instead of 333 convolutions to greatly reduce the amount of computation. Nevertheless, directly processing videos using 3D convolutional networks may result in a larger number of parameters along the temporal dimension, increasing the risk of overfitting. Tran D proposed a similar structure called R(2+1)D. Our proposed method is inspired by TDN and TEA with short- and long-range temporal modeling, taking several continuous frames as input. Our work differs from previous works in that we employ a strategy for long- and short-range temporal modeling to better extract motion information. Although our approach shares similarities with these works, we focus on addressing the problem of spatio-temporal inconsistency (Zheng et al., 2022b). In addition, in the field of autonomous driving, integrating MSN into autonomous systems offers potential advantages for enhancing the environment perception and decision support of the vehicle system. By performing real-time analysis of video and sensor data, MSN can perceive the surrounding environment, accurately recognize the movements of other vehicles, pedestrians, and obstacles, thereby providing autonomous vehicles with richer environmental information. This enables vehicles to more accurately predict the behavior of other traffic participants, thereby improving overall driving safety. However, this application also faces some challenges, especially in terms of real-time requirements, particularly in autonomous driving scenarios that require immediate decision-making. Accurate and efficient action recognition is crucial for rapidly changing traffic environments, making it imperative to address the reduction of algorithm inference time. By incorporating short- and long-range temporal modeling (Wu et al., 2021), our approach aims to enhance the efficiency of action recognition methodologies, showcasing the potential of artificial neural networks in the complex landscape of autonomous system control and decision-making.

## 3 Method

**Figure 1:**
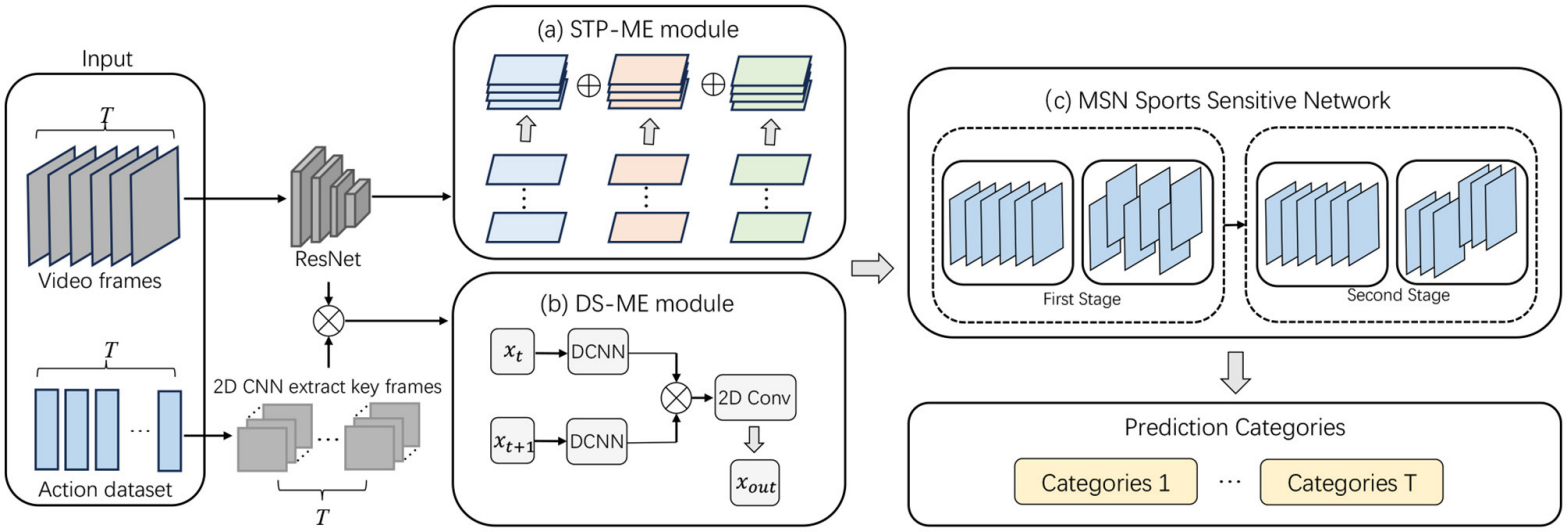
Overall algorithm flowchart. The input section comprises video frames and an action label dataset, where video frames capture the spatiotemporal information of actions, and the action label dataset is used to supervise the model learning process. Subsequently, video frames undergo processing through ResNet and 2D CNN to extract advanced features and key frames, providing a robust foundation for subsequent modules. The STP-ME module addresses the consistency of motion information across different temporal scales using a pyramid structure. This structure enables the module to adaptively handle motion information at different temporal scales, enhancing the model's robustness to scale variations. By applying the pyramid structure to consecutive frames, the STP-ME module focuses on motion information at different temporal scales, including modeling the position and motion rhythm between frames at each pyramid level. The DS-ME module introduces deformable scale convolutions, adaptingively modifying the motion scale of target objects to address unique and irregular motion patterns in dynamic scenes. Adopting multi-scale deformable convolutions covers a broader range of motion, allowing the DS-ME module to intricately model the entire action region and improve modeling accuracy for objects at different scales, particularly when dealing with spatiotemporal inconsistency challenges. Within the overall network architecture, the MSN Sports Sensitive Network's two stages process different levels of feature representations to comprehensively express action information in the video. Finally, the model aggregates multi-category predictions at each time step through Temporal Aggregation, producing the ultimate action classification results.

The overall flowchart of the algorithm in this article is shown in Figure 1:

### 3.1 MSN sports sensitive network

**Figure 2:**
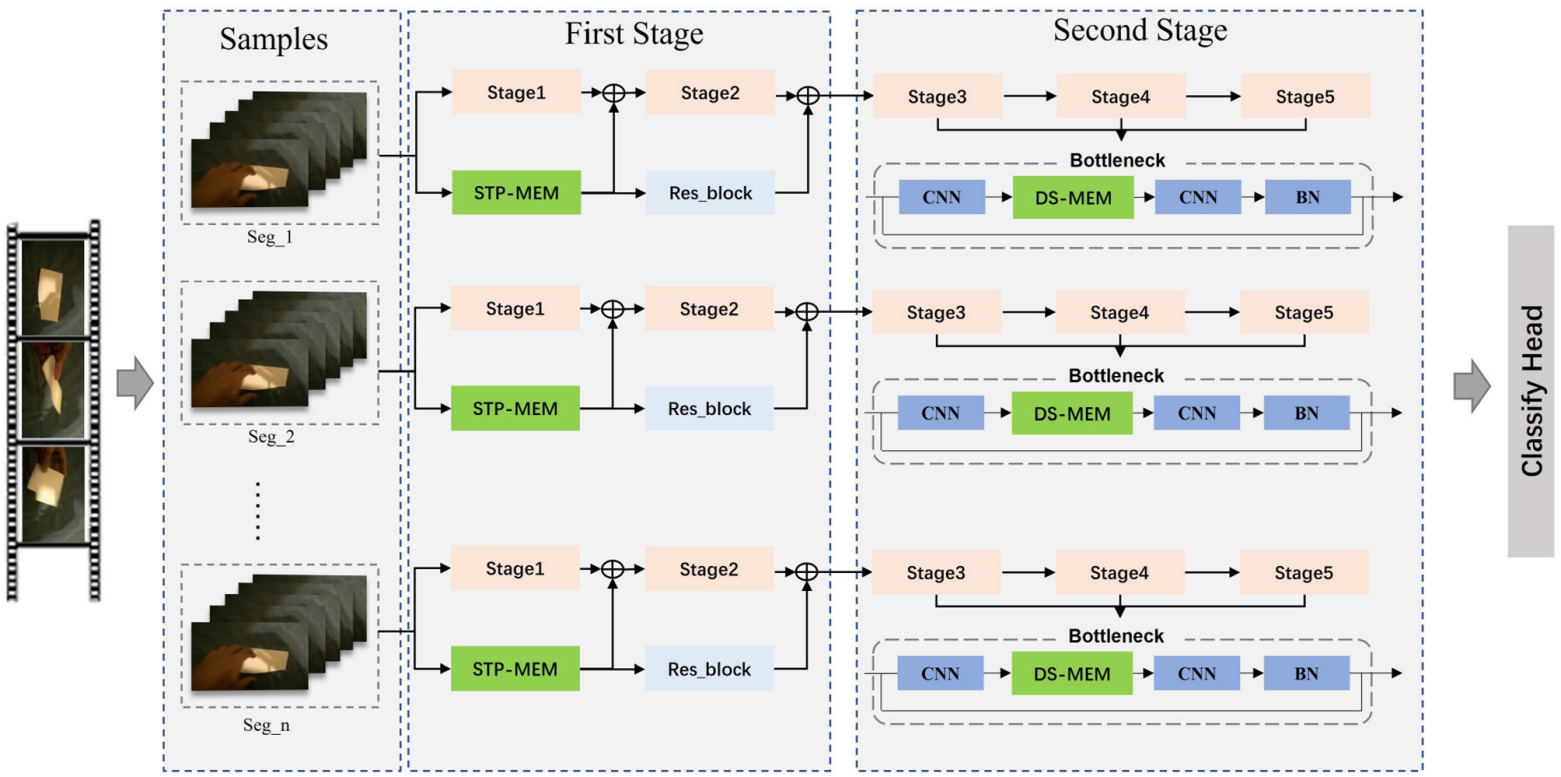
Structure diagram of MSN Sports Sensitive Network. First, input samples containing video frames and corresponding action labels are provided to supervise the model learning process. Proceeding to the First Stage, it is subdivided into Stage1, Stage2, STP-MEM, and Res_block. In Stage1 and Stage2 of the First Stage, advanced feature representations are extracted from the input samples. The STP-MEM module in the First Stage enhances the representation of motion information, adaptively handling motion information at different temporal scales through a pyramid structure, thereby improving the model's robustness to scale variations. Meanwhile, the Res_block strengthens feature propagation through residual connections, helping alleviate the vanishing gradient problem and making the model easier to train. Moving on to the Second Stage, which includes Stage3, Stage4, Stage5, Bottleneck, CNN, DS-MEM, and BN. In each stage of the Second Stage, the model further processes features obtained from the First Stage, gradually forming more abstract and high-level representations. The Bottleneck structure is employed for dimensionality reduction and increased network depth to extract more expressive features. CNN and DS-MEM modules in this stage introduce deformable scale convolutions to better model irregular and unique motion patterns, enhancing modeling accuracy for objects at different scales. BN normalizes features, accelerating convergence, and improving the training stability of the model. Finally, classification is performed through the Classify Head, providing predictions for action categories. The entire flowchart integrates these key components organically, forming a motion-sensitive action recognition network with powerful modeling capabilities for complex motion scenes.

The MSN is a video-level framework that learns action models using entire video information. To improve efficiency, we follow the TSNTSN framework with a sparse and holistic sampling strategy for each video. Our main contribution is to fully consider the scale changes in the space-time dimension when obtaining implicit action information through feature difference and inject this action information into the network in two ways: element-wise addition of the implicit action information extracted by the STP-ME module to the keyframe-wise information extracted by the backbone, and embedding the DS-ME module into the CNN block to increase the processing weight of motion features adaptively. Its structural diagram is shown in Figure 2.

In first stage, each video *V* is divided into T segments of equal duration without overlapping. We randomly sample 5 frames $I^{i}=I_{k-2}^{i},I_{k-1}^{i},I_{k}^{i},I_{k+1}^{i},I_{k+2}^{i}$ from each segment. We select the third frame in as the keyframe and totally obtain *T* key frames $I_{k}=I_{k}^{1},...,I_{k}^{T}$. These keyframes are separate fed into a 2D CNN to extract keyframe-wise features *F* = [*F*_1_, *F*_2_, …, *F*_*T*_]. Besides we applied STP-ME module to extract motion information from the whole 5 frames and supplied it to the original keyframe process pipeline, so as to increase the amount of effective information input and improve the feature's representation power. Specifically, we fuse the keyframe-wise feature and implicit motion 1 information using the following Equation 1:


(1) $$\begin{array}{l} F^{\prime }=F_{i}+S\left(I_{i}\right) \end{array}$$

Where *F*′ denotes the fused feature for segment *i*, *F*_*i*_ is the keyframe-wise feature, *S* denotes our STP-ME module, and it extracts implicit motion information from adjacent frames *I*_*i*_.

In the second stage, we embed the DS-ME module into the CNN block and calculate the channel weight by multiscale cross-segmentation difference. In this way, we could distinguish some feature channels that contain different scales of motion information and enhance these channels to make our net-work pay more attention to the motion. We establish the channel enhance process as follows (Equation 2):


(2) F′=F+D(F)⊙F

Where *D* represents our DS-ME module, *F* is the origin features and *F*′ is the enhanced features. In the current implementation, we only consider adjacent segment-level information for channel weight calculation in each DS-ME module, Details will be described in the following subsections.

### 3.2 STP-ME module

**Figure 3:**
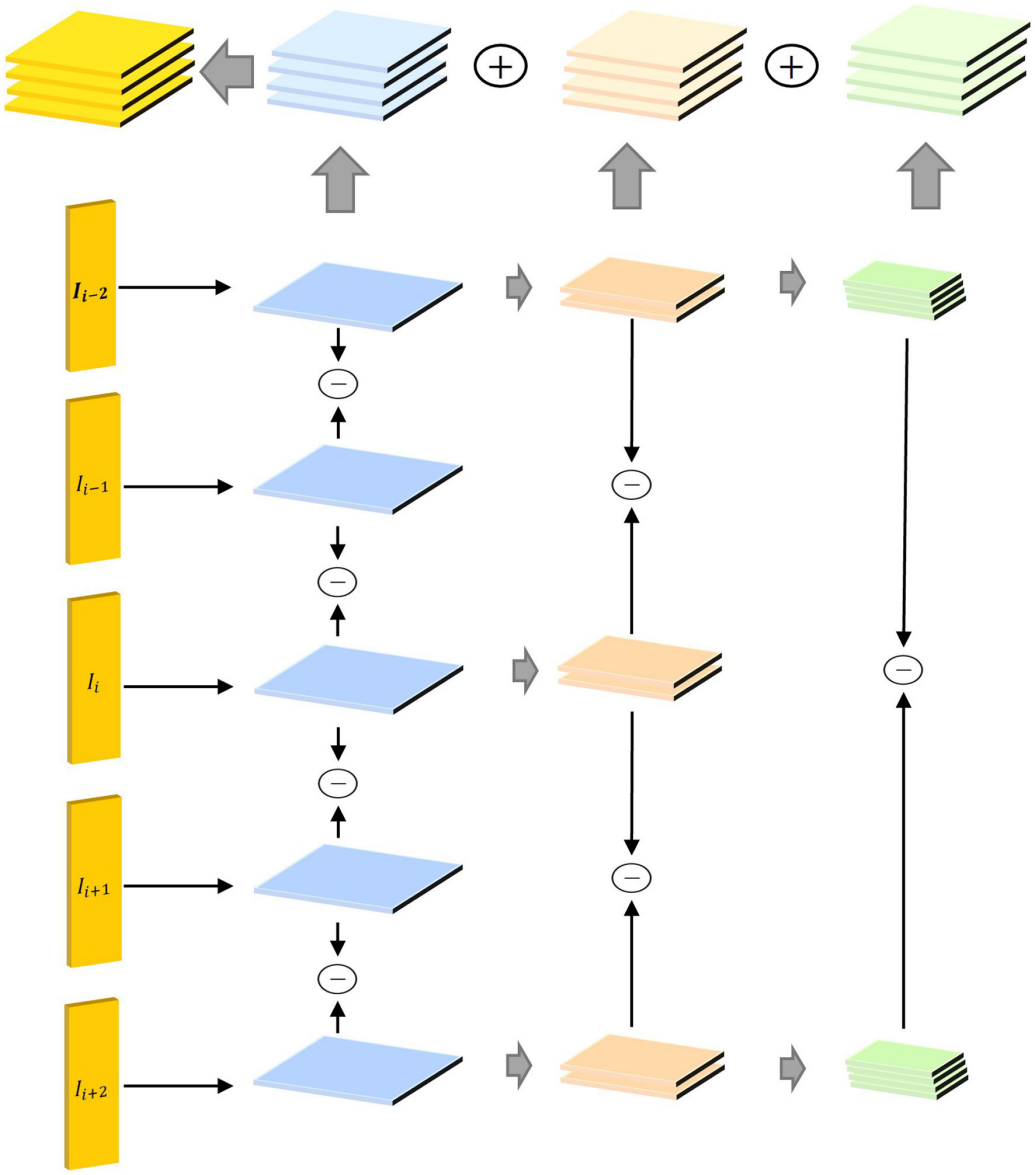
Structure diagram of STP-ME module. The components I_*i*−2_, I_*i*−1_, I_*i*_, I_*i*+1_, and I_*i*+2_ represent frames at the current time step and two preceding and two succeeding time steps, respectively. These frames are introduced as inputs to the STP-ME module, capturing action information in the video at different time steps. Through these inputs, the STP-ME module aims to address the consistency of motion information across various time scales. The primary task of the STP-ME module is to adaptively process motion information at different time scales through a pyramid structure. By applying the pyramid structure between consecutive frames, the module can focus on motion information at different time scales, including modeling the position and motion rhythm between frames. This design enables the STP-ME module to better capture motion information at different time scales, enhancing the overall model's robustness to scale variations.

In a video, the action is reflected in the change of pixel value between adjacent frames. We argue that modest variances across adjacent frames respond well to the nature of the action. Many previous works sample a single frame from a segment which extracts appearance information instead of the motion information contained in each segment. To tackle this problem, we propose the STP-ME module shown in Figure 3.

In STP-ME module, we selected 5 frames in a segment and extracted implicit motion information by feature difference. Furthermore, the time interval often shows a positive correlation with the variance of spatial scale. In specific, as the time interval increases, the spatial scale also increases. Therefore, we aligned the temporal dimension with the spatial dimension from the perspective of scales and extracts implicit motion information from adjacent frames by three steps. Then, make each step corresponds to a different temporal spatial scale.

(1) In the first step, we set the time interval is 1 frame. For each sampled frame *I*_*i*_, we extract several feature differences and then stack them along channel dimension (Equations 3–6):


(3) $$\begin{array}{l} F_{12}=conv1\left(I_{2}\right)-conv1\left(I_{1}\right) \end{array}$$

(4) $$\begin{array}{l} F_{23}=conv1\left(I_{3}\right)-conv1\left(I_{2}\right) \end{array}$$

(5) $$\begin{array}{l} F_{34}=conv1\left(I_{4}\right)-conv1\left(I_{3}\right) \end{array}$$

(6) $$\begin{array}{l} F_{45}=conv1\left(I_{5}\right)-conv1\left(I_{4}\right) \end{array}$$

Where *F*_*ij*_ is feature difference between *I*_*i*_ and *I*_*j*_, *conv*1 is a convolution layer.

(2) At the second step, we set the time interval is 2 frames. We select 3 feature map contains I1, I3, I5 to extract the mid step feature and stack it (Equations 7, 8).


(7) $$\begin{array}{l} F_{13}=conv2\left(I_{3}\right)-conv2\left(conv1\left(I_{1}\right)\right) \end{array}$$

(8) $$\begin{array}{l} F_{35}=conv2\left(I_{5}\right)-conv2\left(conv1\left(I_{3}\right)\right) \end{array}$$

(3) At the third step, we set the time interval is 4 frames. We select 2 feature maps in last step [f1; f5] to extract the final step feature (Equation 9).


(9) $$\begin{array}{l} F_{15}=cons3\left(conv2\left(conv1\left(I_{5}\right)\right)\right)-conv3\left(conv2\left(conv1\left(I_{1}\right)\right)\right) \end{array}$$

(4) Finally, we realize the consistency of each dimension by up-sampling *f*_*u*_ the above features, and fuse them by elementwise addition (Equation 10).


(10) $$\begin{array}{l} F=concat\left(F_{12},F_{12},F_{12},F_{12}\right)+f_{u}\left(concat\left(F_{12},F_{12}\right)\right)+f_{u}\left(F_{15}\right) \end{array}$$

The implicit motion information *F* is fused with the keyframe features, so that the original frame-level representation is aware of motion pattern and able to better describe a segment.

### 3.3 DS-ME module

**Figure 4:**
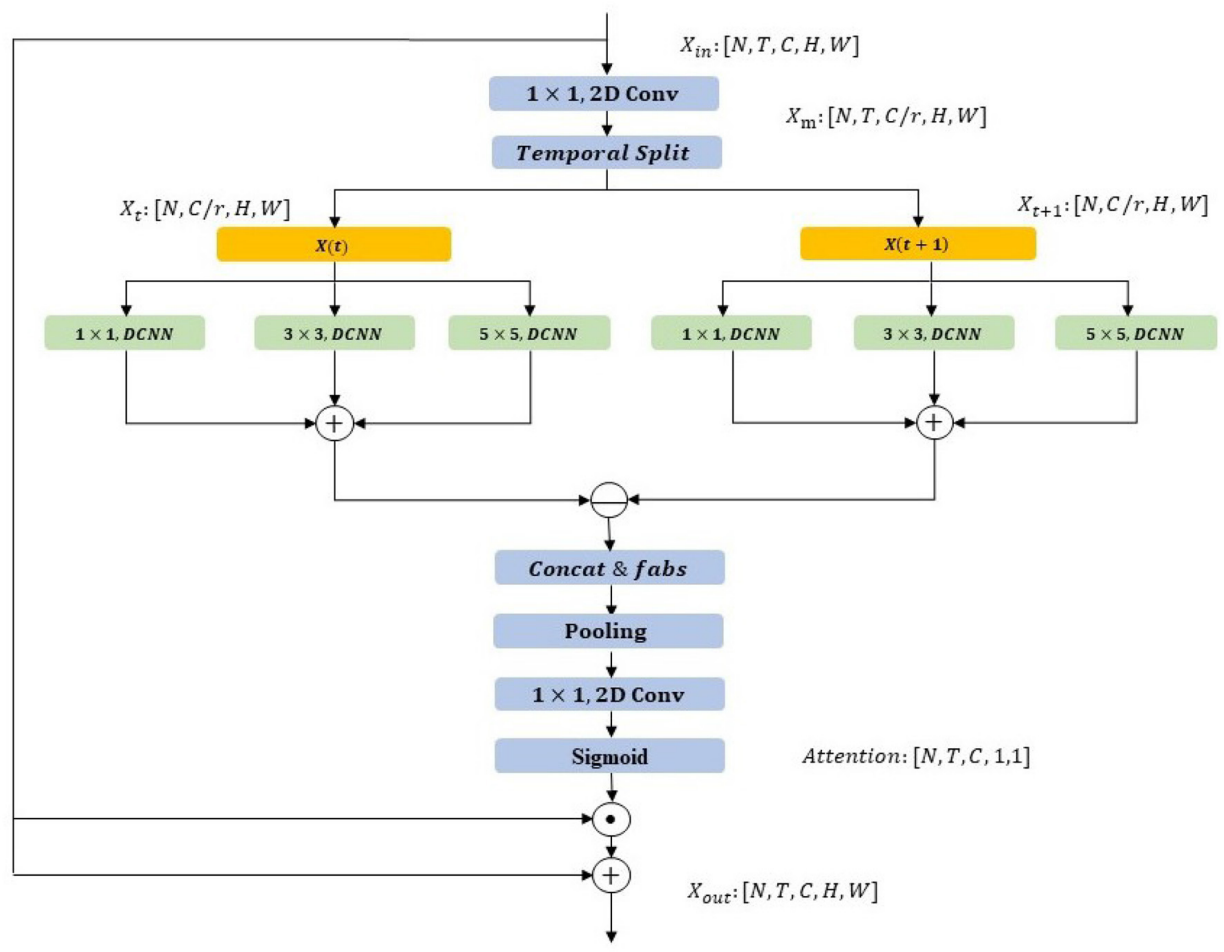
Structure diagram of DS-ME module. The module takes an input tensor [N, T, C, H, W], where N is batch size, T is the number of time steps, and C is the channel dimension. Initial processing involves 1 × 1 and 2D convolutions to adjust channels and extract spatial features, forming a robust foundation for subsequent spatiotemporal modeling. The Temporal Split operation separates the temporal dimension into X(t) and X(t+1), introducing temporal dynamics. These undergo independent 1x1 convolution, deformable convolution (DCNN) with 3x3 kernel, DCNN, and 5 × 5 convolution to intricately model spatiotemporal information, particularly addressing irregular motion patterns. Further operations, including Concat&fabs, pooling, 1 × 1 convolution, 2D convolution, and Sigmoid, fuse and process features, achieving nuanced spatiotemporal modeling. This enhances adaptability to diverse scales and irregular motions, ultimately improving action recognition. The output is a tensor [N, T, C, H, W], representing motion excitation distribution per time step.

The STP-ME module provides a powerful representation for capturing spatial-temporal features, including local motion information within a segment. However, it is essential to leverage this motion information in the second stage to enhance action recognition. While the channel attention strategy has been shown to improve the importance of certain types of information, for action recognition, we need to consider more details. We observe that a complete action is comprised of different scales of motion split and irregular deformations of the action subject in space. To address these issues, we propose the DS-ME module, which employs a multiscale convolution kernel to capture different scales of motion splits and achieve more accurate channel attention calculation. In addition, to smooth the irregular deformation of the action subject, we devise a deformable CNN architecture, as illustrated in Figure 4.

The proposed DS-ME module operates as follows. Firstly, we compress the feature dimension by a ratio of r and split the feature segmentation in the temporal dimension as follows (Equation 11):


(11) $$\begin{array}{l} \left[X_{1},X_{2},...,X_{t}\right]=f_{split}\left(Conv\left(F_{in}\right)\right) \end{array}$$

Where [*X*_1_, *X*_2_, …, *X*_*t*_] is a set of split features in the temporal dimension with a size of *T*, *Conv* is the channel-wise convolution, and *F*_*in*_ is the input feature.

Next, these split features undergo three different scale Deformable CNN (DCNN) operations, namely: (1) a 1 × 1 deformable CNN, (2) a 3 × 3 deformable CNN, and (3) a 5 × 5 deformable CNN. This operation is computed as follows (Equations 12–14):


(12) $$\begin{array}{l} X_{t}^{1}=DCNN_{1}\left(X_{t}\right) \end{array}$$

(13) $$\begin{array}{l} X_{t}^{2}=DCNN_{2}\left(X_{t}\right) \end{array}$$

(14) $$\begin{array}{l} X_{t}^{3}=DCNN_{3}\left(X_{t}\right) \end{array}$$

Where $X_{t}^{1},X_{t}^{2},X_{t}^{3}$ are the deformable features from *X*_*t*_. After that, we could fused $X_{t}^{1},X_{t}^{2},X_{t}^{3}$ and calculate feature difference between consecutive segments as follows (Equation 15):


(15) $$\begin{array}{l} X_{diff}=\left(X_{t+1}^{1}+X_{t+1}^{2}+X_{t+1}^{3}\right)-\left(X_{t}^{1}+X_{t}^{2}+X_{t}^{3}\right) \end{array}$$

where *X*_*diff*_ is the segment-wise feature difference. To avoid the loss of information caused by negative numbers after subtraction, we add an additional absolute value operation *f*_*abs*_ and then perform the maximum value pooling operation *f*_*pooling*_ as follows (Equation 16):


(16) $$\begin{array}{l} W_{\text{raw}}=f_{\text{pooling}}\left(\left|X_{\text{di}}\frac{f}{f^{\prime }}\right|\right) \end{array}$$

where *W*_*raw*_ is the raw weight. To obtain the channel attention weight, we upgrade the channel dimension with a 1x1 convolution *conv* and activate it using the sigmoid function *W*_*raw*_ as follows (Equation 17):


(17) $$\begin{array}{l} W=F_{sig}\left(conv\left(W_{raw}\right)\right) \end{array}$$

Finally, we enhance the video-level representation through a channel attention operation and combine it with the original feature map via a residual connection.

The MSN framework is based on sparse sampling of TSN and operates on a sequence of frames uniformly distributed over the entire video. The framework employs a two-stage motion modeling mechanism that focuses on capturing motion information at different space-time scales. The STP-ME module is inserted in the early stages for fine and low-level motion extraction, while the DS-ME module is used in the latter stages to further strengthen the role of action information in the network. We use a ResNet backbone for the MSN instantiation. Similar to V4D, we use the first two stages of ResNet (also known as the early stage) for implicit motion information extraction within each segment using the STP-ME module. The latter three stages of ResNet (also known as the later stage) are embedded with the DS-ME module for channel attention by capturing different scales of motion splits across segments. To fuse motion information with spatial information in the early stage, we add residual connections between the STP-ME module and the main network for Stage 1 and Stage 2. To enhance the action feature, we embed the DS-ME module to the CNN block and add a channel attention mechanism in each residual block of Stages 3-5.

The pseudocode of the algorithm in this paper is shown in Algorithm 1:

**Algorithm 1:**
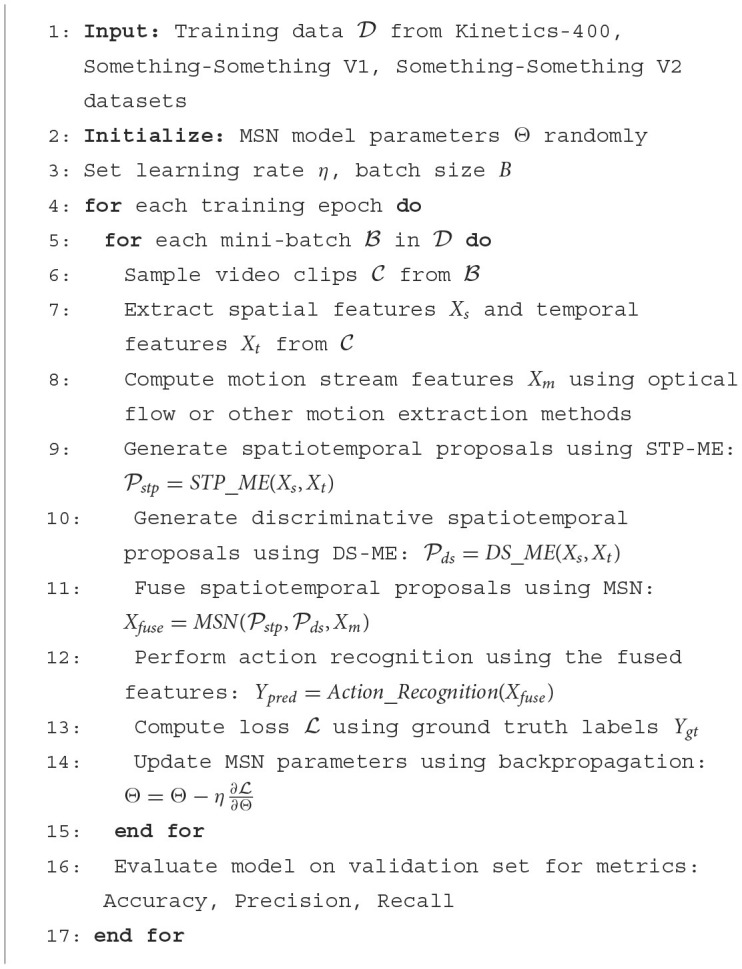
MSN training process.

## 4 Experiment

**Figure 5:**
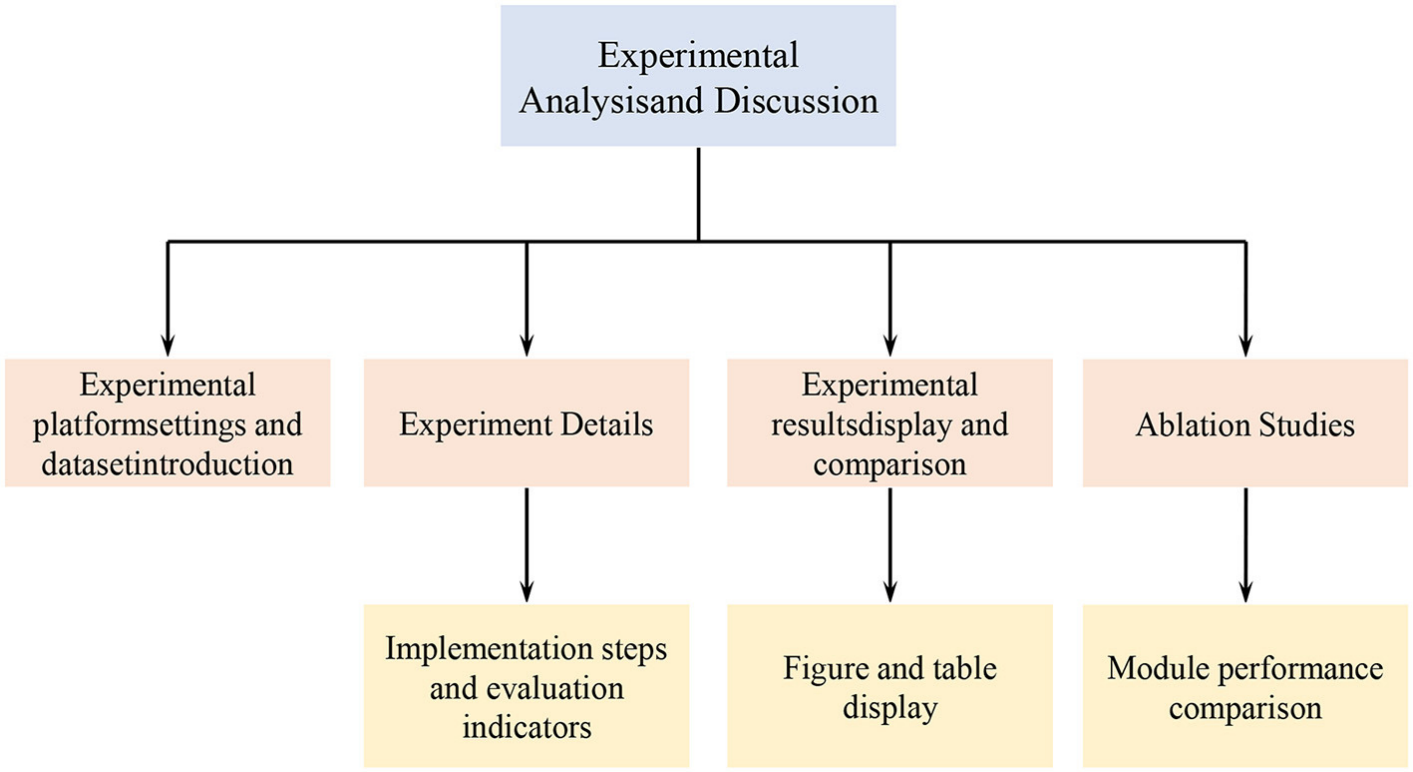
Experimental flow chart.

The experimental flow chart of this article is shown in Figure 5:

### 4.1 Lab environment

- Hardware environment:
  This experiment utilized a high-performance computing server that offers excellent computational and storage capabilities, providing robust support for research on motion-sensitive network action recognition. The server is equipped with an Intel Xeon E5-2690 v4 @ 2.60GHz CPU, a high-performance multi-core processor that delivers substantial computational power suitable for deep learning tasks. With 512GB of RAM, the server ensures abundant memory resources for model training and data processing, contributing to enhanced experimental efficiency. Additionally, the server is outfitted with 8 Nvidia Tesla P100 16GB GPUs, renowned for their outstanding performance in deep learning tasks, significantly accelerating both model training and inference processes.
- Software Environment:
  In this research, we have chosen Python as the primary programming language and PyTorch as the deep learning framework to explore effective methods for motion-sensitive network model. Leveraging the powerful capabilities of deep learning, our objective is to enhance both the performance and efficiency of the model. Taking full advantage of the convenience and flexibility of Python, we rapidly constructed the model. PyTorch, as our preferred deep learning framework, provides us with a rich set of tools and algorithm libraries, significantly streamlining the process of model development and training. With PyTorch's dynamic computation graph mechanism and built-in automatic differentiation functionality, we can more easily build, optimize, and fine-tune the model to achieve superior results in action recognition.

### 4.2 Experimental data

- Kinetics-400 Dataset
  The Something-Something V1 dataset is a video dataset focused on action recognition, renowned for capturing various common actions and object interactions in daily life. The dataset comprises thousands of video clips, with an average duration of around 3 seconds, covering a diverse range of action categories such as stirring, wiping, twisting, and rubbing. Through meticulous annotation, each video clip is explicitly labeled with the ongoing action and involved objects, providing reliable ground truth labels. To collect this diverse data, the dataset's creation leveraged online communities, inviting participants to upload short video clips of themselves performing various actions. This collection method makes the dataset more representative of real-world daily actions, increasing the diversity and complexity of the data. Given the inclusion of many subtle and complex actions, along with diverse interactions between objects and actions, the Something-Something V1 dataset poses a challenge in action recognition tasks. This dataset not only serves as a rich resource for researchers to understand human daily activities but also provides robust support for evaluating model performance in handling fine-grained and multi-category interaction tasks.
- Something-Something V1 Dataset
  The Something-Something V1 dataset stands out as a comprehensive video dataset meticulously crafted for advancing the field of action recognition research. Its distinguishing feature lies in its ability to capture a diverse range of everyday actions and the interactions between individuals and objects, offering valuable insights into human daily activities. With a multitude of action categories, including stirring, wiping, twisting, and rubbing, the dataset encompasses thousands of short video segments, each lasting around 3 seconds. These segments vividly portray a rich variety of actions performed by individuals, contributing to the dataset's diversity. What sets Something-Something V1 apart is its detailed annotation process. Each video segment undergoes careful labeling, providing explicit information about the ongoing action and the objects involved. This meticulous annotation serves as robust ground truth data, essential for training and evaluating action recognition models. The dataset's creation involved a unique approach, leveraging online communities to encourage participants to contribute short video clips featuring diverse actions. This methodology ensures that the dataset captures a more realistic representation of daily activities, adding an extra layer of complexity and authenticity. One of the dataset's notable challenges lies in its inclusion of subtle and complex actions, coupled with diverse interactions between objects and actions. This complexity poses a significant challenge for models aiming to accurately recognize and categorize these nuanced action scenarios.
- Something-Something V2 Dataset
  The Something-Something V2 dataset builds upon the foundation laid by its predecessor, Something-Something V1, and stands as a significant contribution to the realm of action recognition research. Designed to deepen our understanding of human actions, this dataset introduces new challenges and complexities. Something-Something V2 features a diverse array of common actions performed in everyday scenarios, spanning activities such as stirring, wiping, pouring, and more. The dataset comprises a substantial number of video clips, each lasting approximately 3 seconds, offering a rich collection of short segments capturing various actions and interactions. Annotations play a crucial role in Something-Something V2, with meticulous labeling of each video segment specifying the action and involved objects. This detailed annotation serves as invaluable ground truth data for the training and evaluation of action recognition models. What sets Something-Something V2 apart is its introduction of additional challenges, making it more intricate than its predecessor. Notably, the dataset includes actions performed with hands only, pushing the boundaries of action recognition tasks and introducing a new layer of complexity. Intentionally incorporating challenging scenarios, such as ambiguous or subtle actions, Something-Something V2 serves as a benchmark dataset for evaluating the robustness and adaptability of action recognition models.

### 4.3 Experimental comparison and analysis

In this section, we present the experimental results of our MSN framework. Firstly, we describe the evaluation datasets and implementation details. Next, we compare our MSN with state-of-the-art methods. Then, we perform ablation studies to verify the effectiveness of the proposed modules. Finally, we show some visualization results to further analyze our MSN.

In our experiments, we use ResNet50 as the backbone to implement our MSN based on TSN framework, and sample T = 8 or T = 16 frames from each video. For training, each video frame is resized to have the shorter side in [256; 320], and a crop of 224 × 224 is randomly cropped. The total training epoch is set to 100 in the Kinetics dataset and 60 in the Something-Something dataset. We adopt a multi-step learning rate adjustment strategy, where it would be divided by a factor of 10 in each step. In different experiments, our batch size was set to a fixed value of 32. For testing, the shorter side of each video is resized to 256. We implement two kinds of testing schemes: the 1-clip and center-crop, where only a center crop of 224 × 224 from a single clip is used for evaluation, and the 10-clip and 3-crop, where three crops of 256 × 256 and 10 clips are used for testing. The first testing scheme is with high efficiency, while the second one is for improving accuracy with a denser prediction scheme.

We compare our model with state-of-the-art methods including I3D, TAM, GST, SmallBigNet, TEA, and TDN on two benchmarks: Something-Something and Kinetics-400. We report the details used by each method and use the 1 clip and center crop testing scheme for Something-Something and 10 clips and 3 crops for testing on the Kinetics-400 dataset.

**Figure 6:**
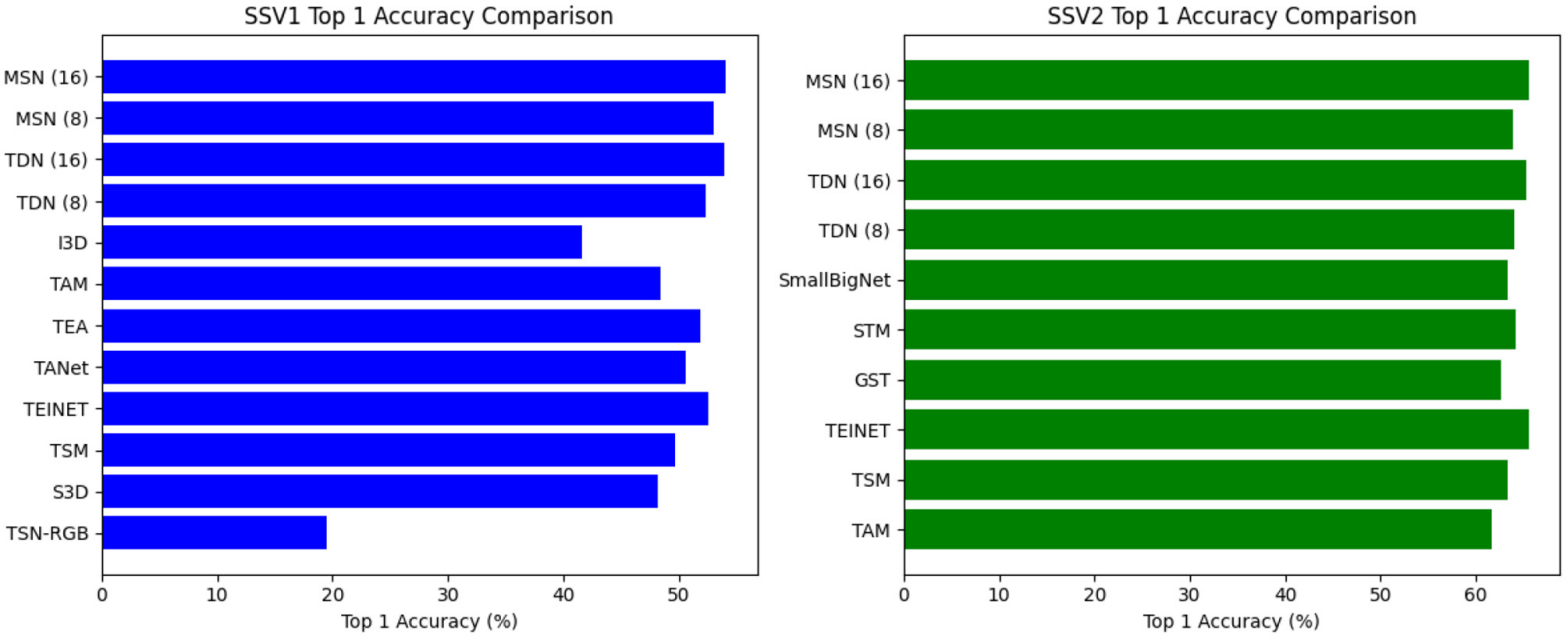
Comparison visualization with state-of-the-art methods on Something-something v1&v2 test set.

Results on something-something. As expected, sampling more frames can further improve accuracy but also increases the FLOPs. We report the performance of both 8-frame MSN and 16-frame MSN. Table 1 shows the comparison results for the proposed MSN on the Something-Something test set, the visualization is shown in Figure 6. Using a ResNet-50 backbone, MSN achieves 53.0% and 54.1% with 8/16 frames, respectively, which are 2.2% and 0.2% better than TEA and TDN, respectively. On the Something-Something v2 dataset, a similar improvement is observed as in SSV1 datasets, especially on 16 frames, which achieved the highest results.

**Table 1:** Comparisons with state-of-the-art approaches on the Something-something v1&v2 test set.

**SSV1(Zhou et al., 2018)**				
**Method**	**Backbone**	**frames**	**Top 1**	**Top 5**
TSN-RGB	BNInception	8	19.50%	-
S3D	Inception	64	48.20%	78.70%
TSM	ResNet50	8+16	49.70%	78.50%
TEINET	ResNet50	8+16	52.50%	-
TANet	ResNet50	8+16	50.60%	-
TEA	ResNet50	16	51.90%	80.30%
TAM	bLResNet50	16	48.40%	-
I3D	ResNet50	32	41.60%	72.20%
TDN	ResNet50	8	52.30%	80.60%
TDN	ResNet50	16	53.90%	82.10%
MSN	ResNet50	8	53.00%	81.50%
MSN	ResNet50	16	54.10%	82.30%
**SSV2 (Materzynska et al., 2020)**				
**Method**	**Backbone**	**frames**	**Top 1**	**Top 5**
TAM	bLResNet50	16*2	61.70%	88.10%
TSM	ResNet50	16*6	63.40%	88.50%
TEINET	ResNet50	8+16	65.50%	89.80%
GST	ResNet50	16	62.60%	87.90%
STM	bLResNet50	16*30	64.20%	89.80%
SmallBigNet	ResNet50	8+16	63.30%	88.80%
TDN	ResNet50	8	64.00%	88.80%
TDN	ResNet50	16	65.30%	89.50%
MSN	ResNet50	8	63.90%	89.20%
MSN	ResNet50	16	65.50%	89.90%

**Figure 7:**
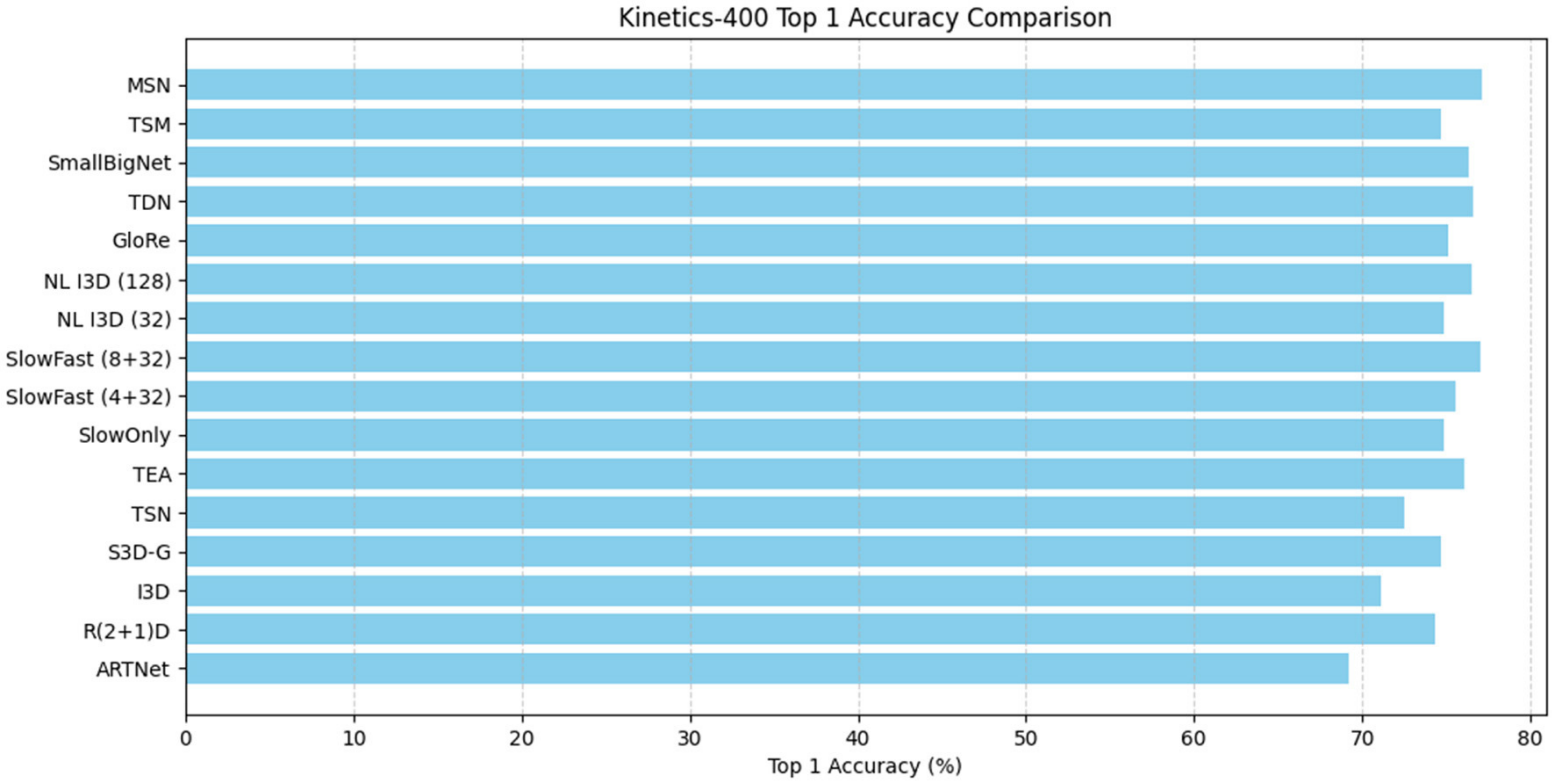
Comparative visualization of Kinetics-400 test system and state-of-the-art methods.

Results on kinetics. On Kinetics-400, we compare our MSN with other state-of-the-art methods. We note that these are comparisons of systems which can differ in many aspects. Nevertheless, our method surpasses all existing RGB or RGB+flowbased methods by a good margin. Without using optical flow and without any bells and whistles, Table 2 shows our model achieved the best performance of 77.1%, the visualization is shown in Figure 7.

**Table 2:** Comparisons with state-of-the-art approaches on the Kinetics-400 test set.

**Kinetics-400(Carreira et al., 2018)**					
**Method**	**Backbone**	**frames**	**GFLOPs**	**Top 1**	**Top 5**
ARTNet	R18	16	23.5	69.20%	88.30%
R(2+1)D	R34	16	152	74.30%	91.40%
I3D	Inception	64	108	71.10%	89.30%
S3D-G	Inception	64	71.4	74.70%	93.40%
TSN	Inception	25	16	72.50%	90.20%
TEA	R50	16	70	76.10%	92.50%
SlowOnly	R50	8	41.9	74.90%	91.50%
SlowFast	R50	4+32	36.1	75.60%	92.10%
SlowFast	R50	8+32	65.7	77.00%	92.60%
NL I3D	R50	32	N/A	74.90%	91.60%
NL I3D	R50	128	282	76.50%	92.60%
GloRe	R50	8	28.9	75.10%	N/A
TDN	R50	8	36	76.60%	92.80%
SmallBigNet	R50	8	57	76.30%	92.50%
TSM	R50	16	65	74.70%	N/A
MSN	R50	8	36.2	77.10%	93.10%

We present the results of our experiments to verify the effectiveness of the proposed STP-ME and DS-ME modules, using ResNet50 as the backbone and evaluating the model's accuracy on the something-to-something v1 dataset.

**Figure 8:**
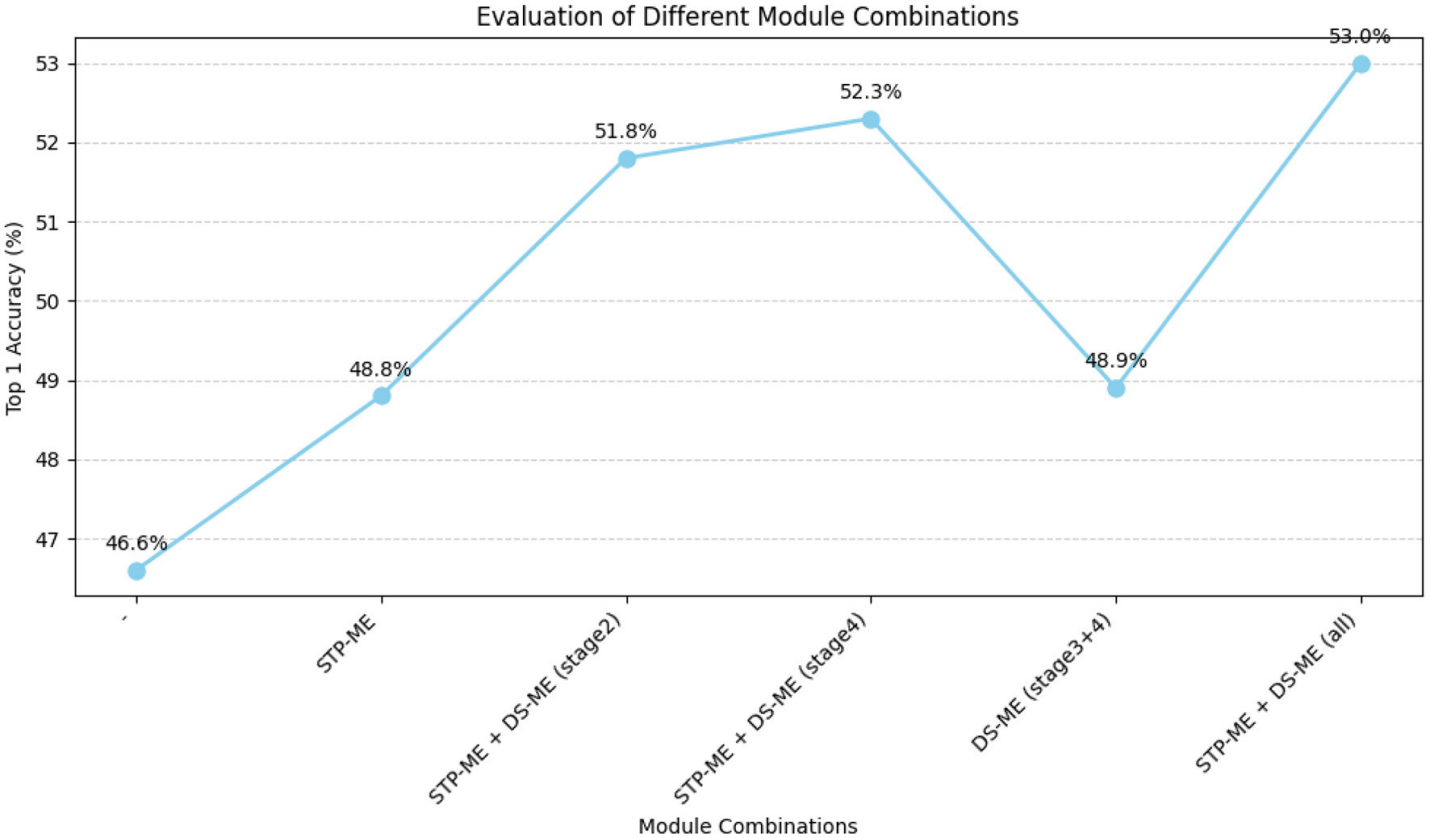
Evaluation comparison visualization of four different combinations.

Study on the effect of STP-ME module and DS-ME module. To investigate the impact of the STP-ME and DS-ME modules, we conducted a comparative study and evaluated four different combinations, as summarized in Table 3, the visualization is shown in Figure 8. First, we established a baseline network without any of these modules, which achieved an accuracy of 46.6%. Then, we separately added the STP-ME module and the DS-ME module to the early layers of the network. As the number of STP-ME modules increased, the accuracy improved, achieving 48.8% and 51.8%, respectively. Similarly, the DS-ME module improved the baseline accuracy by 2.3%, achieving an accuracy of 48.8%. Finally, we included all usable modules in our final model, which achieved the best performance of 52.3% and 53.0% on the something-to-something v1 dataset.

**Table 3:** Evaluation of four different combinations.

**STP-ME module**		**DS-ME module**			**Top 1**
**stage1**	**stage2**	**stage3**	**stage4**	**stage5**	
					46.60%
✓					48.80%
✓	✓				51.80%
✓	✓	✓	✓		52.30%
		✓	✓	✓	48.90%
✓	✓	✓	✓	✓	53.00%

In addition, we compared our STP-ME module with similar modules from other works, including S-TDM proposed in TDN and the super image proposed in StNet (as shown in Table 4). From the results, we found that the super-image module could increase the top-1 accuracy by 2%, and S-TDM could increase it by 4.9%. However, our STP-MEM achieved the maximum performance gain of 5.3%.

**Table 4:** Performance comparison of STP-ME module and other modules.

**Fusion mode**	**GFLOPs**	**Top 1**
Concatation		52.30%
Element-wise average		52.10%
Element-wise addition		53.00%

In our study on the STP-ME module, we found that the fusion operation of different scale features is a crucial step. Therefore, we compared different fusion operations of the STP-ME module, including (1) channel concatenation, (2) element-wise addition, and (3) element-wise average. As shown in Table 5, the element-wise addition achieved the best accuracy of 53.0%, while the element-wise average and channel concatenation obtained top-1 accuracies of 52.3% and 52.1%, respectively. We note that the action information captured by different scale operators is complementary, and therefore the performance of the feature can be maximized when only element-wise addition is used.

**Table 5:** Comparison of ablation experiments of STP-ME modules.

**Similar module**	**Model**	**dataset**	**Top 1 (Increase)**
Super-Image	St-Net	Kintics-600	2%
S-TDM	TDN	SSV1	4.90%
STP-MEM	MSN	SSV1	5.30%

**Figure 9:**
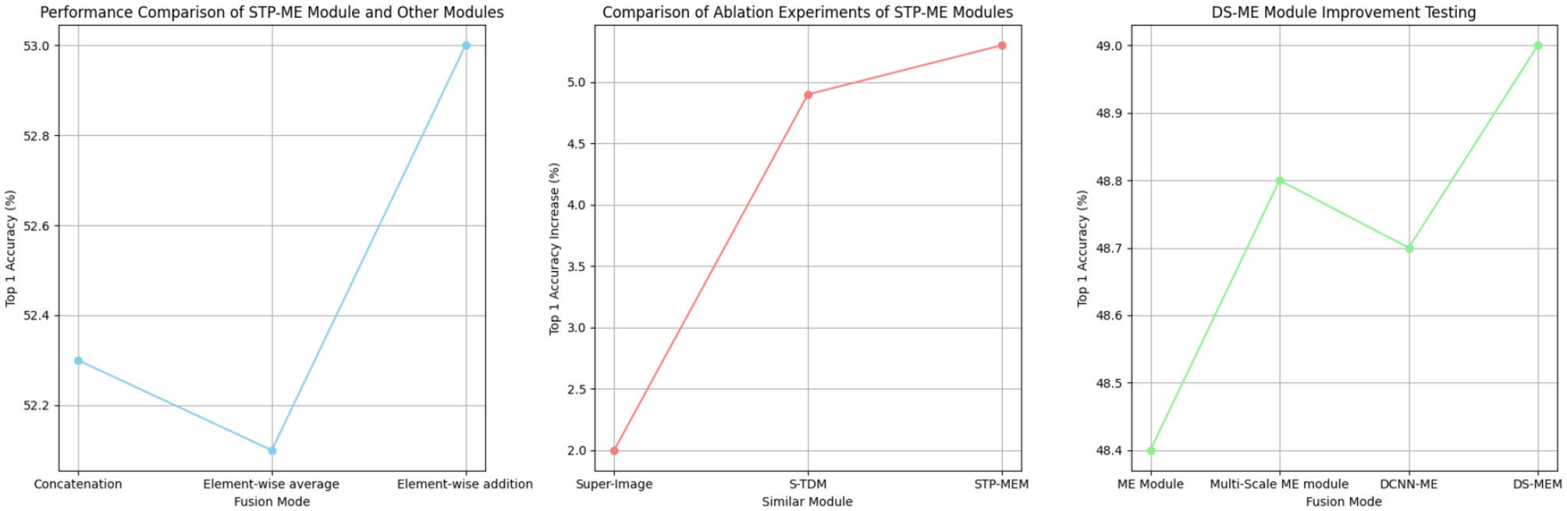
Comparative visualization of module performance and ablation experiments.

Furthermore, we conducted a study on the DS-ME mod- ule, where we made several improvements to the deficiencies present in the ME modules of the previous TEA. We tested these improvements one by one, including four networks: (1) using ME modules, (2) using multi-scale ME modules, (3) using DCNN ME modules, and (4) using DS-ME module. As shown in Table 6, these improved modules provided performance improvements of 0.2%, 0.4%, and 0.5%, respectively, the visualization is shown in Figure 9.

**Table 6:** DS-ME module improvement testing.

**Fusion mode**	**GFLOPs**	**Top 1**
ME module		48.40%
Multi-scale ME module		48.80%
DCNN-ME		48.70%
DS-MEM		49.00%

**Figure 10:**
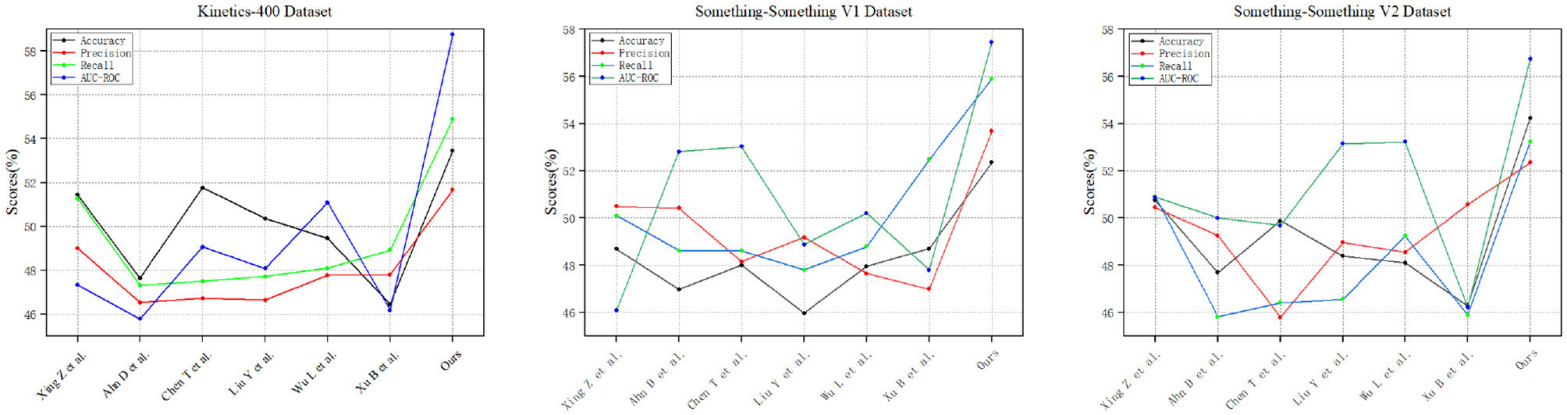
Comparison of experimental indicators between this method and other methods on three data sets.

**Figure 11:**
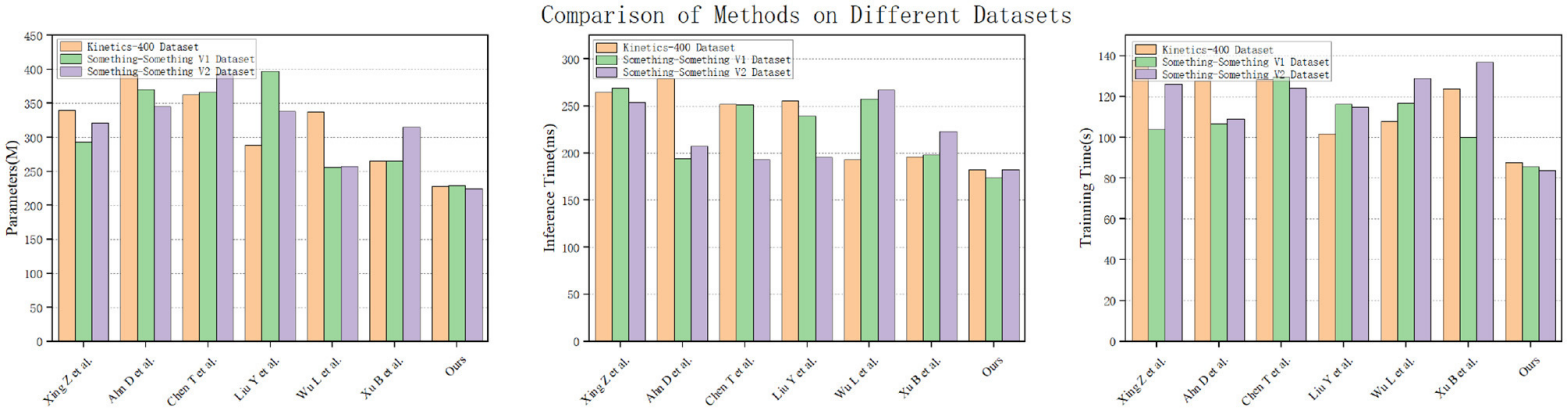
Experimental comparison of parameters, inference time and training time of this method with other methods on three datasets.

Figure 10 shows the performance metrics of various models, including Xing Z et al., Ahn D et al., Chen T et al., Liu Y et al., Wu L et al., Xu B et al., and “Ours,” evaluated across three distinct datasets: Kinetics-400, Something-Something V1, and Something-Something V2. Notably, our model, labeled as “Ours,” consistently outshines the others across all datasets, boasting the highest accuracy, precision, recall, and AUC-ROC values. Then, Figure 11 provides a comprehensive overview of various models, including Xing Z et al., Ahn D et al., Chen T et al., Liu Y et al., Wu L et al., Xu B et al., and “Ours,” assessed across three different datasets: Kinetics-400, Something-Something V1, and Something-Something V2. The models' performance is evaluated based on three key parameters: the number of parameters (in millions), inference time (in milliseconds), and training time (in seconds). Notably, our model, labeled as “Ours,” stands out with the lowest number of parameters, efficient inference times, and remarkably short training durations across all datasets. Specifically, on the Kinetics-400 dataset, “Ours” exhibits a competitive parameter count (227.64 M), efficient inference time (182.46 ms), and notably quick training time (87.62 s). This trend continues across the Something-Something V1 and V2 datasets, reinforcing the efficiency of our model in terms of model complexity, real-time inference, and training speed compared to other evaluated models.

**Figure 12:**
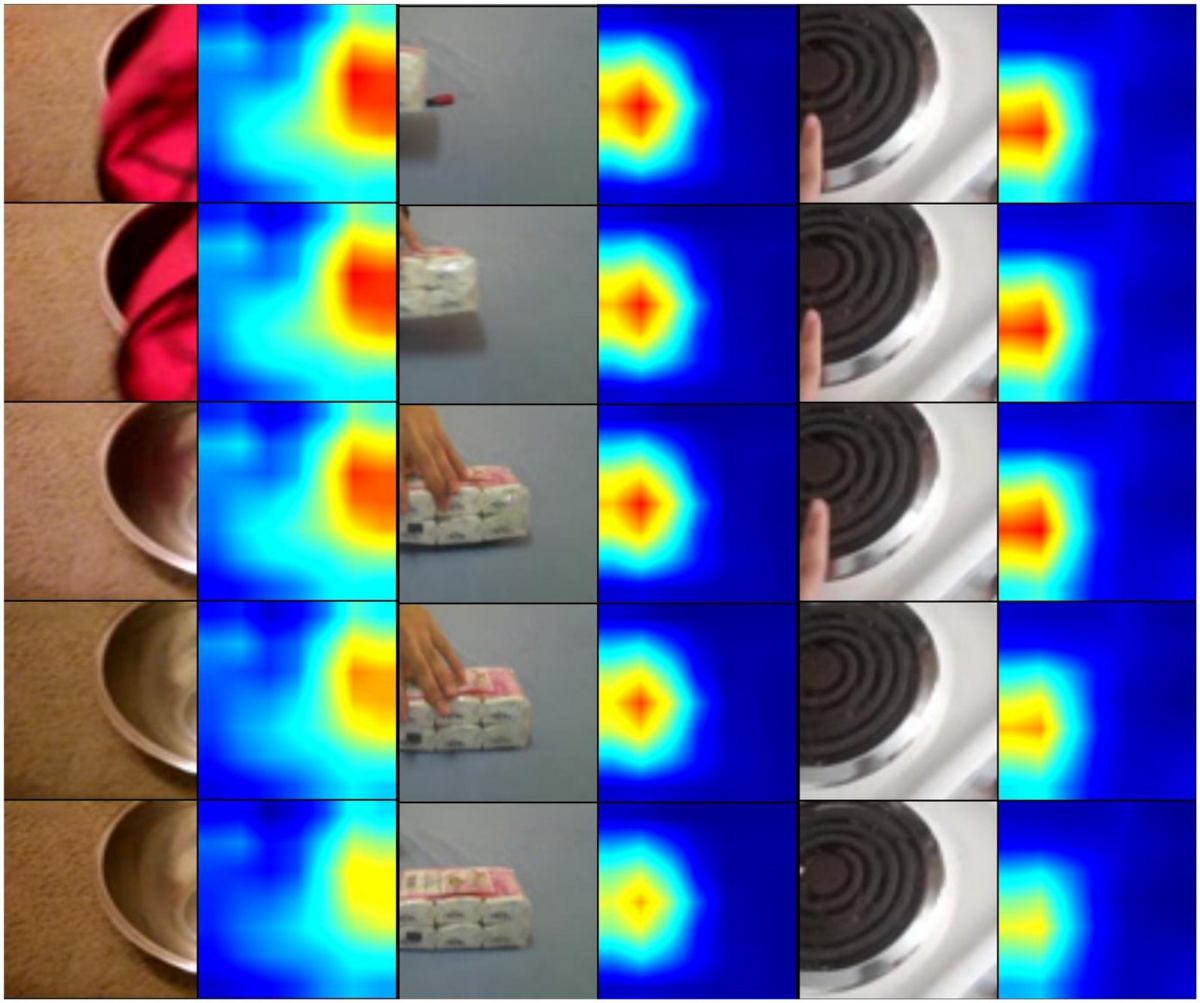
Visualization of activation maps with Grad-CAM++.

We visualize the class activation maps with Grad-CAM++ (Chattopadhay et al., 2018) and results are shown in Figure 12. Specifically, we used 8 frames as input and only visualized the activation maps in the center frames. The visualization results clearly demonstrate that the baseline method with only temporal convolutions cannot effectively focus on motion-salient regions, while our proposed MSN with the STP-ME module and DS-ME module for motion modeling is able to more accurately localize action-relevant regions.

## 5 Discussion

The experimental results demonstrate the effectiveness of the introduced STP-ME and DS-ME modules, marking a significant advance in the field of spatiotemporal modeling for action recognition. The research focuses on the theoretical foundations and practical applications of artificial neural networks (ANN) in autonomous system control and decision-making, and our experimental results bring valuable insights. The quantitative evaluation of MSN against state-of-the-art methods on Kinetics-400 and Something-Something datasets reveals compelling results. Achieving an accuracy of 77.1% on Kinetics-400, MSN outperforms existing RGB or RGB+flow-based methods by a significant margin. This demonstrates not only the theoretical effectiveness of the proposed method but its practical superiority in large-scale action recognition benchmarks. The experiments involving different frame sampling rates (8-frame MSN and 16-frame MSN) showcase the scalability of MSN in handling varied input scenarios. While using more frames generally improves accuracy, MSN maintains competitive performance even with a reduced frame sampling rate. This scalability is crucial for applications where computational resources are limited. Ablation studies offer detailed insights into the impact of module additions. The step-wise improvement in accuracy with the introduction of the STP-ME and DS-ME modules provides a clear understanding of their individual contributions. This data-driven analysis substantiates the claim that these modules are not merely additions but essential components for enhancing action recognition performance. The comparative analysis of fusion operations within the STP-ME module provides nuanced information on the best strategy for integrating multiscale features. The superior performance of element-wise addition in achieving an accuracy of 53.0% underscores its effectiveness in preserving and maximizing valuable information across different temporal scales. When compared with similar modules from previous works, such as S-TDM and super image modules, the STP-ME module exhibits the highest performance gain of 5.3%. This data-driven comparison quantifies the advancements achieved by MSN in capturing intricate motion information, setting it apart as a leading method for spatial-temporal modeling. Beyond accuracy, the evaluation of MSN's efficiency in resource utilization is critical. The balance achieved between accuracy and computational efficiency, particularly with the sparse sampling strategy and two-stage motion modeling mechanism, positions MSN as a practical solution for real-world applications where both accuracy and efficiency are paramount. MSN's consistent performance across diverse datasets, such as Kinetics-400 and Something-Something, highlights its ability to generalize well to various action recognition scenarios. This generalization is a key characteristic, indicating the adaptability and versatility of MSN in handling different types of actions, scales, and temporal variations. This adaptability aligns with the requirements of autonomous systems, which often encounter diverse action types, scales, and temporal variations. In summary, the theoretical implications of MSN in enhancing spatial-temporal modeling, coupled with its practical performance and efficiency, resonate with the goals of advancing artificial neural networks within the realm of autonomous system control and decision-making. The identification of specific scenarios where MSN excels opens avenues for future optimizations, ensuring its robustness and applicability in specialized application domains within autonomous systems.

## 6 Conclusion

In this paper, we present a novel network architecture for action recognition, called MSN. Our approach is both simple and effective, and involves leveraging multiple temporal rates in actions using the temporal pyramid module, which captures motion information at different scales by adjusting the size of the convolution kernel and time interval simultaneously. Additionally, we introduce a new motion excitation module that employs a multi-scale deformable CNN to adjust the motion scale of the target object, which is often non-uniform and irregular. We evaluate our method on four challenging datasets, namely Something-Something V1, Something-Something V2 and Kinetics-400, and compare our results to those of other state-of-the-art (SOTA) approaches. The results demonstrate that MSN performs exceptionally well in a variety of challenging scenarios. The theoretical foundation of MSN is in line with the continuously evolving landscape of spatiotemporal modeling, resonating with the broader discussions about the integration of Artificial Neural Networks (ANN) in autonomous system control and decision-making. Its adaptability across datasets and scenarios, coupled with efficiency, positions MSN as a promising tool that not only advances action recognition but also contributes to the theoretical and practical foundations of ANN in the autonomous systems domain. While MSN demonstrates commendable performance in action recognition, it is essential to acknowledge its computational cost, interpretability challenges, and the need for further extension to new environments. Future developments should prioritize enhancing the interpretability of MSN, achieving real-time adaptability, exploring transfer learning in diverse environments, delving into human interaction understanding, and seamlessly integrating MSN into autonomous systems. We can design network structures with enhanced interpretability, introduce attention mechanisms, or employ visualization techniques to illustrate the model's key steps and rationale during decision-making. Additionally, domain adaptation, transfer strategy design, improving model robustness, and incorporating online learning mechanisms are also indispensable aspects to consider. These steps will pave the way for establishing a more robust, transparent, and versatile network, aligning with the ongoing developments in the field of artificial neural networks within autonomous system control and decision-making.

## Data availability statement

The original contributions presented in the study are included in the article/supplementary material, further inquiries can be directed to the corresponding author.

## Author contributions

JG: Conceptualization, Data curation, Funding acquisition, Investigation, Methodology, Project administration, Resources, Software, Validation, Writing – original draft. YY: Data curation, Methodology, Project administration, Resources, Software, Visualization, Writing – review & editing. QL: Conceptualization, Data curation, Funding acquisition, Project administration, Writing – review & editing.
